# Oxidative Stress in the Murine Model of Extraparenchymal Neurocysticercosis

**DOI:** 10.3390/microorganisms12091860

**Published:** 2024-09-08

**Authors:** Diego Generoso, Tatiane de Camargo Martins, Camila Renata Corrêa Camacho, Manuella Pacífico de Freitas Segredo, Sabrina Setembre Batah, Alexandre Todorovic Fabro, Edda Sciutto, Agnès Fleury, Pedro Tadao Hamamoto Filho, Marco Antônio Zanini

**Affiliations:** 1 Botucatu Medical School, UNESP—Universidade Estadual Paulista, Botucatu 18618-686, Brazil; 2 Ribeirão Preto Medical School, USP—Universidade de São Paulo, Ribeirão Preto 14049-900, Brazil; 3 Instituto de Investigación Biomédicas, UNAM—Universidade Nacional Autónoma de México, Ciudad de México 04510, Mexico; 4 INNN—Instituto Nacional de Neurología y Neurocirurgía, Ciudad de México 14269, Mexico

**Keywords:** neurocysticercosis, oxidative stress, malondialdehyde, protein carbonyl, inflammation

## Abstract

Oxidative stress is associated with several infectious diseases, as well as the severity of inflammatory reactions. The control of inflammation during parasite destruction is a target of neurocysticercosis treatment, as inflammation is strongly related to symptom severity. In this study, we investigated the presence of malondialdehyde and protein carbonyl, two by-products of reactive oxygen species (ROS), in an experimental model of extraparenchymal neurocysticercosis. Twenty male and twenty female rats were inoculated with 50 cysts of *Taenia crassiceps* in the subarachnoid space of the cisterna magna. Ten animals (five males and five females) were used as controls. Three months after inoculation, their brains were harvested for oxidative stress and histological assessments. Infected animals had higher scores for inflammatory cell infiltrates, malondialdehyde, and protein carbonyl. These results encourage future efforts to monitor oxidative stress status in neurocysticercosis, particularly in the context of controlling inflammation.

## 1. Introduction

Neurocysticercosis (NCC) is a common parasitic disease of the central nervous system (CNS) in low- and middle-income countries. Despite a recent decrease in its endemicity (particularly in Latin America), NCC remains a relevant public health problem with significant social and economic burdens in endemic countries [1,2,3]. NCC is also a health challenge in countries receiving immigrants from endemic regions, although its extent and impact remain unclear [4,5].

NCC may present as one of two different forms depending on the site at which the larvae of *Taenia solium* are found within the CNS: parenchymal (P-NCC) and extraparenchymal (EP-NCC). While P-NCC is highly responsive to clinical treatment, with a relatively benign course (except for massive infection), EP-NCC is less responsive to anthelminthic drugs and is more prone to complications such as vasculitis, hydrocephalus, and increased intracranial pressure [6,7,8]. Inflammation is a key pathophysiological mechanism underlying such complications [9], and oxidative stress has been shown to increase the severity of NCC, primarily in patients with neurocysticercosis-induced hydrocephalus [10]. Oxidative stress is initiated by neutrophil and monocyte activation during inflammation. When activated, these cells release reactive oxygen species (ROS), such as superoxide radicals, hydrogen peroxides, and hydroxyl radicals. Excess ROS, known as oxidative stress, damages cells and tissues by inducing oxidative damage to DNA, proteins, and lipids, resulting in organ dysfunction [11]. Lipids are the primary macromolecules affected by oxidative stress, resulting in toxic products such as malondialdehyde, which can destroy proteins via a mechanism termed protein carbonylation [12]. Oxidative stress and inflammation are involved in the pathophysiology of neurocysticercosis.

Since this mechanism still requires better understanding, the use of experimental models of NCC has allowed for a better understanding of NCC pathophysiology and opened pathways for testing new therapeutic regimens [13,14,15,16,17]. The experimental model of EP-NCC with *Taenia crassiceps* has further been shown to closely resemble the features of human diseases, including brain parenchymal displacement, hydrocephalus, and subarachnoid inflammation [18,19]. Previously, *T. crassiceps* was not considered as a suitable model for studying neurocysticercosis, because the parasite does not invade the brain parenchyma—rather, it displaces the nervous tissue. This concern would make sense for studies focusing on parenchymal neurocysticercosis [13]. Nevertheless, for EP-NCC, the growth of the parasite with the displacement of the brain is precisely what is desired. Therefore, the use of *T. crassiceps* reproduces the human disease and allows for the investigation of several pathophysiologic mechanisms, such as oxidative stress.

In the present study, we aimed to investigate the presence of in situ oxidative stress markers in EP-NCC rats, as well as to compare differences between male and female infected rats.

## 2. Materials and Methods

In this study, we used female and male Wistar rats (*Rattus norvegicus*) handled according to the ethical guidelines and current legislation for animal research. This project was approved by the local Institutional Review Board for ethics of animal use (CEUA 1330/2019).

Rats aged 6 weeks were housed under 12 h light–dark cycles at room temperature (approx. 21 °C), with free availability of water and conventional commercial food. In total, 50 animals (25 males and 25 females), including 10 rats as controls (5 of each sex), were used. The sample size was estimated considering a mortality rate of 30% following the inoculations and a rate of 66% of successful infections, based on reported data on oxidative stress in neurocysticercosis, with a type II error of 20%, and a level of significance of 5%.

### 2.1. Study Design

**Figure 1:**
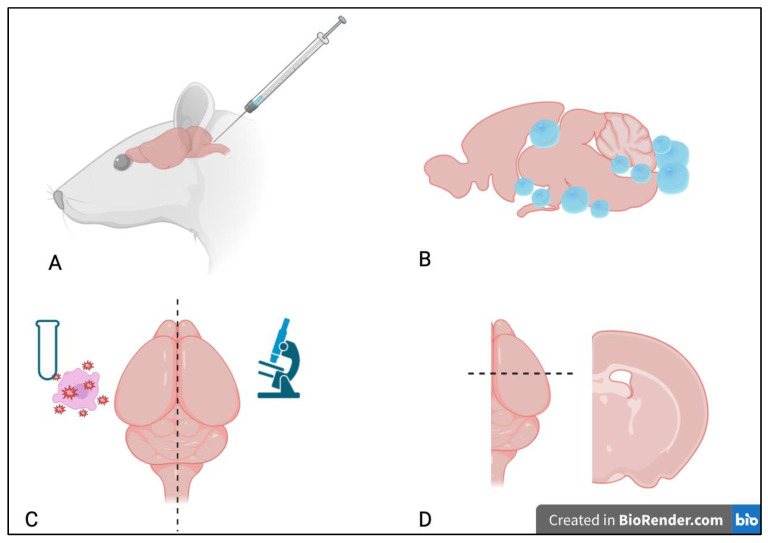
Schematic representation of the experimental procedures. (**A**): The cyst injection was performed at the cisterna magna, i.e., the subarachnoid space between the posterior-inferior portion of the cerebellum and the dorsal portion of the medulla. (**B**): Three months after the inoculation, the cysts could be observed throughout the subarachnoid space, located predominantly in the basal convexity of the brain and brainstem. (**C**): The right hemisphere was used for histologic analysis, and the left hemisphere for oxidative stress essays. (**D**): The dorsal and coronal views at the level of tissue histologic assessment.

A total of 40 rats (20 males and 20 females) received a subarachnoid injection of 50 cysts of *T. crassiceps* (Figure 1A), and were kept alive for three months post-inoculation. After the observational period, the animals were euthanized with a thiopental overdose, and their brains were harvested following wide craniectomy. The presence of cysts in the subarachnoid space upon brain harvesting confirmed the inclusion of the animal (Figure 1B), while rats that did not present with any cysts (not infected) were discarded. The right hemisphere was embedded in 4% buffered paraformaldehyde, and the left hemisphere was frozen and stored at −80 °C for posterior quantification of oxidative stress markers (malondialdehyde and protein carbonyl). Ten animals (five male and five female) did not receive any intervention and underwent the same protocol for euthanasia and tissue processing at the same age as the inoculated animals (Figure 1C).

### 2.2. Parasites and Inoculations

Cysts of *T. crassiceps* (ORF strain) were maintained in the peritoneal cavity of mice (*Mus musculus)*, as described elsewhere [20,21], and aseptically transferred onto Petri dishes with saline 0.9%. Cysts were selected based on their size (0.5 mm) and membrane integrity. Each rat received 50 cysts in 0.2 mL of saline 0.9%, as previously described [16]. Briefly, after intraperitoneal anesthesia with xylazine and ketamine, the skin was incised at the posterior cervical region along the craniocervical junction, and the subarachnoid space of the cisterna magna was punctured with a 24-gauge needle for injection. Finally, the skin was sutured with a 4.0 mononylon thread.

### 2.3. Histologic Assessment

After 24 h of fixation in 4% buffered paraformaldehyde, the right hemispheres were dehydrated with increasing concentrations of alcohol, clarified in xylene, and paraffinized. Slices with 5 µm of thickness were subsequently taken at the level of the optic chiasm (Figure 1D) and stained with hematoxylin and eosin.

The lymphocyte infiltrates in the basal arachnoid–pia–mater–brain interface were analyzed semi-quantitatively using the following score: 1, absence of lymphocyte infiltration; 2, discrete infiltration (scarce and sparce lymphocytes); 3, moderate infiltration (focally present lymphocytes); and 4, intense infiltration (several lymphocytes diffusely).

### 2.4. Oxidative Stress Assessment

Because free radicals (reactive oxygen species) are rapidly degraded, they cannot be measured directly; instead, their activity must be measured indirectly by assessing their byproducts. Herein, we assessed the malondialdehyde and protein carbonyl levels. Malondialdehyde is a derivative of the peroxidation of polyunsaturated fatty acids [22], and carbonyl proteins refer to the process by which the oxidation of the side chains of amino acids leads to the formation of reactive ketones and aldehydes [23].

After defrosting the left hemisphere, the samples were homogenized. For malondialdehyde quantification, 250 µL of brain tissue supernatant was mixed with 750 µL of 10% trichloroacetic acid for protein precipitation. The samples were then centrifuged at 3000 rpm for 5 min (Eppendorf ^®^ Centrifuge 5804-R, Hamburg, Germany), and the supernatant was removed. Thiobarbituric acid (TBA) was added in a proportion of 0.67% (1:1) and the samples were heated for 15 min at 100 °C. After cooling, the absorbance was measured at 535 nm using a Spectra Max 190 microplate reader (Molecular Devices, Sunnyvale, CA, USA). The MDA concentration was calculated using the molar extinction coefficient (1.56 × 10^5^ M^−1^ cm^−1^) and the absorption of the samples, with the final result reported in nmol/g of protein.

To assess protein carbonyl, 100 µg of brain tissue was homogenized in ice-cold PBS solution (1 mL, pH 7.4) using an ULTRA-TURRAX^®^ T25 basic (IKA^®^ Werke, Staufen, Germany) and then centrifuged at 800× *g* for 10 min at 4 °C. The supernatant was incubated with 2,4-dinitrophenylhydrazine (DNPH) for 1 h to derivatize carbonyl proteins. The samples were subsequently deproteinized with trichloroacetic acid and centrifuged. The supernatant was discarded, and the sediment was washed three times with an ethanol/ethyl acetate solution to remove excess DNPH, preventing the quantification of chromophores not bound to carbonyl protein. Finally, 6M guanidine hydrochloride was added to the washed sediment, and the absorbance was measured at a wavelength of 370 nm. The total protein concentration in the samples was quantified using commercial assays to adjust the carbonyl protein values. The concentrations of carbonyl proteins were expressed as µg/mg of protein.

### 2.5. Statistical Analysis

The data were analyzed in three steps. First, all infected animals (regardless of sex) were compared to the control group. For the parametric data, we used an independent *t*-test. Next, the same tests were used to compare the infected males and females with their respective controls. Finally, we compared infected male and female rats. The analyses were then performed using the Statistical Package for the Social Sciences (SPSS) version 24.0 (IBM Corp., Armonk, NY, USA) and GraphPad Prism v. 8.2.0 (GraphPad Software, La Jolla, CA, USA). Differences were considered statistically significant at *p* < 0.05.

## 3. Results

Among the twenty male rats, seven died after inoculation and two did not have any cysts, while among the twenty female rats, eight died and two did not develop infection. Therefore, 11 male and 10 female infected rats were included in the final analysis and compared to 10 controls (5 males and 5 females).

**Figure 2:**
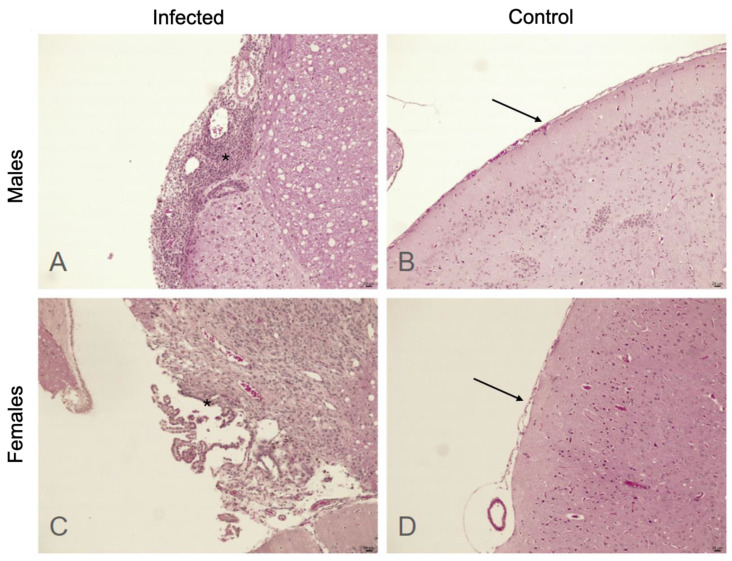
Microscopic view of the basal arachnoid–pia–mater–brain interfaces for male (**A**,**B**) and female (**C**,**D**) rats. In control animals, a thin arachnoid layer (arrows) juxtaposed to the pia–mater with preserved histoarchitecture (**B**,**D**) can be observed. In infected animals (**A**,**C**), the arachnoid is thickened and lymphocytes (*) can be observed in intense (**A**) or discrete (**C**) infiltrations.

Histologic assessment revealed a normal arachnoid–pia–mater–brain interface in the control animals, with a thin and delicate arachnoid layer juxtaposed to the pia–mater, with few detectable lymphocytes. Among the infected animals, variable degrees of lymphocyte infiltration, with many cases of thickened arachnoids and lymphocytes in the foci of infiltration or diffuse distribution, were observed (Figure 2). The median lymphocyte infiltration score was higher in the infected animals than in the control animals (3.0 vs. 1.0, *p* < 0.001). This difference remained significant between males (*p* = 0.038) and females (*p* = 0.003). We found no significant differences between the infected male and female rats (*p* = 0.721).

**Figure 3:**
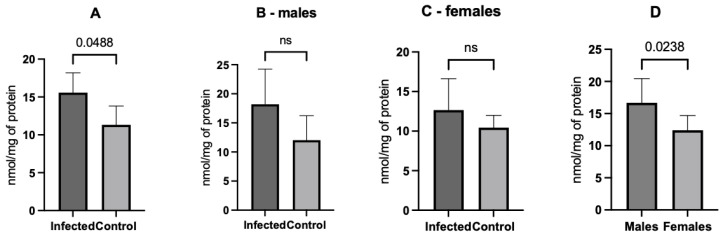
Comparisons of the malondialdehyde concentration between the experimental groups. When considering all infected animals, the mean concentration was higher than the control animals (**A**). However, the differences were no longer significant (ns) when analyzing male (**B**) and female (**C**) animals with their controls. Further, considering the infected animals, males had a higher concentration of malondialdehyde than females (**D**).

Figure 3A shows a significantly higher mean of malondialdehyde levels (15.6 ± 5.8 nmol/mg) in the infected than in the control rats (11.3 ± 3.2 nmol/g), (*p* = 0.048). Malondialdehyde levels showed no significant difference between males (Figure 3B, *p* = 0.059) and females (Figure 3C, *p* = 0.305). Infected males had higher MDA concentrations than females (*p* = 0.024, Figure 3D).

**Figure 4:**
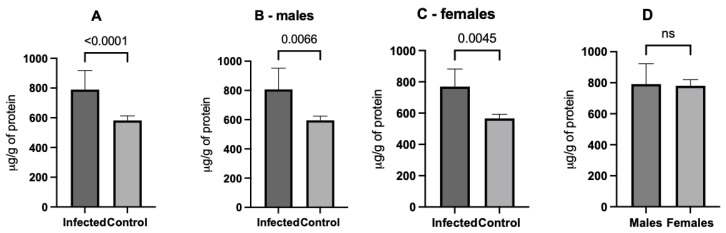
Comparisons of protein carbonyl between the experimental groups. The infected animals presented higher concentrations when comparing all animals (**A**), the males (**B**), and females (**C**) with their respective controls. There was no difference between infected males and females (**D**). ns: non-significant.

Infected animals had a higher concentration of protein carbonyl than controls (789.2 ± 128.7 vs. 582.5 ± 30.3 µg/g, *p* < 0.001); this effect was also seen when comparing only male (*p* = 0.007) and female rats (*p* = 0.005) (Figure 4). No significant differences were observed between the infected males and females (*p* = 0.513).

## 4. Discussion

Inflammation occurs throughout the natural history of NCC, even in cases of calcified cysts [9]. Inflammation plays a pivotal role in effective parasite destruction; however, this effect is a double-edged sword, as it is also related to symptom severity. Since the use of anthelminthics can trigger inflammation by increasing the exposure of parasites to immunological surveillance, the concomitant use of corticosteroids is necessary to control the possible increase in severity [24]. As such, the control of inflammation without impairing the effectiveness of cysticides is a significant research target.

Reactive oxygen and nitrogen species produced during inflammation may damage DNA, proteins, and lipids, resulting in consequent lesions in the surrounding cells and tissues [25]. While physiological amounts of these reactive species can be tolerated under healthy conditions, oxidative stress has been clearly demonstrated to impair neurological function under several conditions, such as neurodegenerative and infectious diseases [26,27,28].

Few studies have addressed the oxidative stress status in patients with NCC. Rodríguez et al. found that patients with NCC had higher levels of lipid peroxidase (LP) in the cerebrospinal fluid than controls, while the LP levels were also significantly correlated with the inflammatory response and severity of the symptoms [12]. Interestingly, even patients receiving steroid therapy exhibited increased LP levels. Prasad et al. studied children with NCC and found higher levels of malondialdehyde, protein carbonyl, and nitrite, accompanied by low levels of antioxidants in the CSF [29]. It has also been reported for different infections that such acute respiratory conditions caused by different viruses where oxidative stress is implicated with lung tissue injury and epithelial barrier dysfunction could also increase the susceptibility to new infections. Increased oxidative stress may also promote tissue fibrosis and metabolic dysfunction, as has been reported in viral hepatitis [30].

Oxidative stress in the brain has been evaluated in animal models of meningitis. Barichello et al. found increased levels of protein carbonyl in the hippocampus and the cortex from 6 to 96 h after the subarachnoid injection of group B Streptococcus [31]. Using a similar approach with *Escherichia coli*, Giridharan et al. also observed increased levels of MDA, protein carbonyl, and superoxide dismutase in the hippocampus [32]. The oxidative stress in the hippocampus may be related to epileptogenesis, and the use of antioxidant drugs may improve long-term outcomes of patients exposed to potential epileptogenic insults, such as traumatic brain injury [33,34]. Additionally, for chronic infections such as NCC, the scenario may be less promising. For instance, in experimental models of toxoplasmosis, the use of antioxidants is associated with increase in the parasitemia [35].

Another possible effect associated with oxidative stress in our experimental model is hydrocephalus, which refers to an active distension of the ventricular system of the brain from an imbalance on CSF production, circulation, and absorption [36]. Hydrocephalus is a common feature of EP-NCC due to a combination of inflammation (triggered by the host response against the parasite) and mechanical obstruction of the CSF pathways (caused by the presence of the cysts in the narrow points of the ventricle and basal cisterns) [7]. We have previously demonstrated that this model leads to hydrocephalus [18], and several experimental models of hydrocephalus have demonstrated that oxidative stress occurs and is related to the severity of neurological lesions [37,38,39].

In the present study, we demonstrated the occurrence of oxidative stress in a murine model of extraparenchymal neurocysticercosis. The differences between infected and control animals were even more pronounced in protein carbonyl levels, while significant differences were maintained even between male and female rats. Further studies should attempt cyst preservation upon brain harvesting to correlate the intensity of oxidative stress with the parasite load.

In previous studies, female rats showed greater inflammatory features than male rats [40,41]. In contrast, in the present study, we found higher concentrations of malondialdehyde in males. A possible explanation for this relationship between hormones and the modulation of the antioxidant defense system; estrogen and progesterone display antioxidant properties [42]. However, females seem to present more intense inflammatory features in NCC than males [43,44]. The crosstalk between cysticercosis, sex hormones, and oxidative stress should be further explored.

The results obtained in our study should be considered to optimize the treatment of ExP-NCC and reduce associated collateral damage.

In summary, in this study, we found significant evidence to indicate an increase in oxidative stress markers in NCC, one more feature consolidating the similarities between this model of extraparenchymal neurocysticercosis and human infection. Monitoring the oxidative stress status throughout the course of infection could provide relevant information regarding the optimal time to administer anti-helminthics and corticoids, as well as for the withdrawal of corticoids. However, further experimental studies are required to evaluate this possibility from a translational perspective.

## Data Availability

The data presented in this study are available on request from the corresponding author. The data are not publicly available due to privacy recommendations.
